# Padding for Splints

**Published:** 1900-01

**Authors:** 


### Padding for Splints.

Absorbent cotton does not make good padding for splints. Ordinary cotton batting is far more elastic and will not gradually pack together by absorbing moisture from the skin.—*Richmond Journal of Practice.*

